# Genome-wide identification and expression analysis of the *ADH* gene family in *Artemisia annua* L. under UV-B stress

**DOI:** 10.3389/fpls.2025.1533225

**Published:** 2025-03-19

**Authors:** Hengyu Pan, Peiqi Shi, Shan Zhong, Xiaoxia Ding, Shengye Bao, Siyu Zhao, Jieting Chen, Chunyan Dai, Danchun Zhang, Xiaohui Qiu, Baosheng Liao, Zhihai Huang

**Affiliations:** ^1^ The Second Clinical College, Guangzhou University of Chinese Medicine, Guangzhou, China; ^2^ College of Life Science and Technology, Mudanjiang Normal University, Mudanjiang, China; ^3^ School of Chinese Materia Medica, Tianjin University of Traditional Chinese Medicine, Tianjin, China

**Keywords:** alcohol dehydrogenases, *Artemisia annua*, gene duplication, expression patterns, UV-B stress, qRT-PCR

## Abstract

*ADHs* are key genes that catalyze the interconversion between alcohols and aldehydes, which play crucial roles in plant adaptation to a range of abiotic stresses. However, the characterization and evolutionary pathways of *ADH* genes in the antimalarial plant *Artemisia annua* are still unclear. This study identified 49 *ADH* genes in *A. annua* and conducted a detailed analysis of their structural features, conserved motifs, and duplication types, revealing that tandem and dispersed duplications are the primary mechanisms of gene expansion. Evolutionary analysis of *ADH* genes between *A. annua* (*AanADH*) and *A. argyi* (*AarADH*) revealed dynamic changes, with 35 genes identified deriving from their most recent common ancestor in both species. *ADH1*, crucial for artemisinin production, had two copies in both species, expanding via dispersed duplication in *A. annua* but whole-genome duplication in *A. argyi*. CREs and WGCNA analysis suggested that *AanADH* genes may be regulated by UV-B stress. Following short-term UV-B treatment, 16 DEGs were identified, including *ADH1* (*AanADH6* and *AanADH7*), and these genes were significantly downregulated after two hours treatment (UV2h) and upregulated after four hours treatment (UV4h). The expression changes of these genes were further confirmed by GO enrichment analysis and qRT-PCR experiments. Overall, this study comprehensively characterized the *ADH* gene family in *A. annua* and systematically identified *AanADH* genes that were responsive to UV-B stress, providing a foundation for further research on their roles in abiotic stress responses.

## 1 Introduction

*Artemisia annua* L., a traditional Chinese medicine belonging to Asteraceae family, is the main source for artemisinin, a compound widely used in the global treatment of malaria (Bora and Sharma, 2011; Ma et al., 2018). Extensive studies have revealed that *A. annua* can produce diverse plant secondary metabolites (PSMs), including alcohols, esters, aldehydes, phenols and terpenes (Soni et al., 2022). These PSMs are multifunctional compounds that play crucial roles in plant growth and defense mechanisms (Bennett and Wallsgrove, 1994; Anjali et al., 2023; Divekar et al., 2022). In plants, the prominent functions of PSMs are to provide protection against stresses such as pathogens, insects, and predators (Erb and Kliebenstein, 2020). Additionally, some PSMs can mitigate abiotic stresses including flooding, salinity, reactive ROS and UV radiation (Nakabayashi and Saito, 2015; Anzano et al., 2022; Salam et al., 2023). For instance, in salvia plants, the ROS scavenging system was induced by diterpene to enhance drought tolerance (Yadav et al., 2021). Recent advancements have shown that external stimuli, as iron oxide nanoparticles (Fe3O4 NPs), can further enhance the production of artemisinin in *A. annua* by upregulating the key genes in the terpenoid biosynthetic pathway and inducing oxidative stress (Ayoobi et al., 2024). Therefore, elucidating the regulatory roles of key genes in *A. annua*’s secondary metabolic pathways could provide crucial insights into how this medicinal plant coordinates its defense systems against both biotic and abiotic challenges.

Alcohol dehydrogenase (ADH, alcohol: NAD oxidoreductase, EC 1.1.1.1), is a zinc-binding enzyme dimer widely distributed in various organisms. It relies on NAD (P) cofactors to interconvert ethanol and acetaldehyde (and other short linear alcohol/aldehyde pairs) (Strommer, 2011). The *ADH* gene family is extensive and can be classified into three main subfamilies based on their chain length: short-chain dehydrogenase/reductase (SDR)-ADH (~250 amino acid residues), medium-chain dehydrogenase/reductase (MDR)-ADH (∼350 amino acid residues) and long-chain dehydrogenase/reductase (LDR)-ADH (600 to 750 amino acid residues or about 385 to 900 amino acid residues) (Alka et al., 2013; Jörnvall et al., 2013). Structurally, ADHs are Zn-binding enzymes that possess two conserved domains: a GroES-like (ADH_N) and a zinc-binding (ADH_zinc_N) domain (Taneja and Mande, 1999; Jörnvall et al., 2010). Presently, the MDR-ADH subfamily is most prevalent in plants, with its members typically containing zinc ligands in the active site (Nordling et al., 2002; Hedlund et al., 2010).

The *ADH* gene family plays vital roles in plant growth, development and stress responses (Strommer, 2011), which help plants adapt to various environmental stresses including flooding (Bailey-Serres and Voesenek, 2008), drought (Hu et al., 2022), cold (Davik et al., 2013), salt (Hu et al., 2022), and the exogenous hormone abscisic acid (ABA) (Yi et al., 2017), etc. In *Arabidopsis*, *ADH* expression can be induced by hypoxia, dehydration, low temperature and the phytohormone ABA (Dolferus et al., 1994). *ScADH3* appeared to enhance cold tolerance in sugarcane by regulating ROS-related genes to maintain ROS homeostasis (Su et al., 2020). Similarly, in melon, *CmADHs* exhibited tissue-specific expression pattern and responded to various hormonal stresses (Jin et al., 2016). Among these diverse functions, the hypoxic or anaerobic response was the most well-known and oldest role for *ADH* genes in plants (Strommer, 2011). For example, the transcript levels of *ZmAdh1* and *ZmAdh2* in maize both increased rapidly under low oxygen conditions and then decreased under anaerobic conditions (Andrews et al., 1993). Beyond stress responses, *ADH* is also involved in the biosynthesis of aromatic compounds in plants, catalyzing the selective conversion of short linear aldehydes and alcohols into aromatic precursors (Thompson et al., 2007; Manríquez et al., 2006; Zeng et al., 2020). While *ADH1* in *A. annua* has been functionally characterized as a key catalyst in artemisinin biosynthesis by catalyzing the dehydrogenation of artemisinic alcohol to artemisinic aldehyde (Feng et al., 2021; Liao et al., 2022b), the broader functional landscape of *ADH* gene family in this medicinal species remains largely unexplored.

While these functional insights highlight *ADH*’s multifaceted roles across plant species, systematic characterization within *Artemisia* remains limited. Although *ADH1* is implicated in artemisinin biosynthesis, the evolutionary dynamics and functional diversification of *ADH* in this medicinally crucial genus remain unclear. This knowledge gap persists despite available genomic resources for key species like *A. annua* and *A. argyi*, where research has predominantly focused on isolated gene characterization or cross-species homology-based predictions. The recent advancement in haplotype-resolved genome assembly for *A. annua* enables comprehensive analysis of *ADH* gene paralogs, evolutionary patterns, and transcriptional network. Here, we identified 49 *AanADH* genes through bioinformatics analyses, characterizing their chromosome distribution, structural characteristics, duplication events, and conducting comparative evolutionary analyses with *A. argyi*. We further combined RNA-seq and qRT-PCR to examine the expression patterns of the *AanADH* genes following short-term UV-B across three timepoints (0/2/4 h). This multi-omics investigation provided fundamental insights into *AanADH* functions and reference for in-depth studies on the role of the *ADH* genes in abiotic stress responses in *A. annua*.

## 2 Materials and methods

### 2.1 Plant material

The tissue culture seedlings of the *A. annua* LQ-9 strain were collected for *ADH* genes expression analysis. The tender leaves of LQ-9 strain were cultured in the growth medium (MS medium 4.43 g/l + Sucrose 30 g/l + Agar 7 g/l + 6-BA 0.5 mg/l + NAA 0.06 mg/l) for 7 days, and then transplanted to the rooting medium (MS 2.215 g/l + Sucrose 30 g/l + Agar 7 g/l + IBA 0.5 mg/l + 0.1 mg/l NAA). The pH value of both media was 6.0. The materials were cultivated in a plant incubator, with daytime conditions set at 10 h, 3000 lx, 80% humidity, and 25 °C. *A. argyi* was collected from Nanyang City, Henan Province, China, which was stored at -80 °C refrigerator for subsequent PCR amplification experiment.

### 2.2 Identification of *ADH* genes

The genome of *A. annua* LQ-9 haplotype 0, and the transcriptome from different tissues of *A. annua* were downloaded from the Global Pharmacopoeia Genome Database (GPGD, http://www.gpgenome.com/) (Liao et al., 2022a, 2022b). The annotated protein of LQ-9 haplotype 0 genome was searched against the PFAM database (Pfam 32.0) using PfamScan (E value ≤ 1e-5) (http://www.ebi.ac.uk/Tools/pfa/pfamscan). Genes with hits to ADH_N domain (PF08240) or ADH_zinc_N domain (PF00107) were considered as the candidate *ADHs*. Next, to avoid missing gene models of *ADHs*, candidate *ADHs* and publicly available protein sequences from NCBI NR database (https://www.ncbi.nlm.nih.gov/protein) of *A. annua* with the above domains (PF08240 and PF00107) were used to search against LQ-9 haplotype 0 genome using BLASTP (e-value = 1e−5). Then, all the candidate *ADHs* were viewed and manually corrected using the Apollo browser (version 2.3.1) (Dunn et al., 2019), and genes that met the following criteria were retained: (1) complete gene structure supported by RNA-seq/Iso-Seq data or (2) the presence of both PF08240 and PF00107 domains. This study utilized the haplotype genomes HAP1 and HAP2 of *A. argyi* from the GPGD database, and the *A. thaliana* TAIR10.1 genome assembly and annotation files retrieved from NCBI (https://ftp.ncbi.nlm.nih.gov/genomes/all/GCF/000/001/735/GCF_000001735.4_TAIR10.1/). The identification of *ADH* gene family in these species followed the same bioinformatics pipeline previously established for *A. annua*.

### 2.3 Physicochemical and structural characteristics of ADH proteins

Physiochemical characteristics of ADH proteins were analyzed using the ExPASY ProtParam tool (https://web.expasy.org/protparam/) (Gasteiger et al., 2003). Subcellular localization of ADH proteins were predicted using the online tool Cell-PLoc2.0 (http://www.csbio.sjtu.edu.cn/bioinf/Cell-PLoc-2/) (Chou and Shen, 2010). The conserved domain of ADH proteins was detected by the InterProt (https://www.ebi.ac.uk/interpro/, accessed on 1 April 2024) (Paysan-Lafosse et al., 2023). Conserved motif annotations were obtained using the MEME (version 5.5.5) (https://meme-suite.org/meme/tools/meme/, accessed on 3 April 2024) (Bailey et al., 2015).

### 2.4 Collinearity, gene duplication and cis-regulatory elements analysis of *ADH* genes

The One Step MCScanX integrated into the TBtools 2.083 (Chen et al., 2023a) was used to identify the synteny relationships and duplication patterns of *ADH* genes within and between species. Gene collinearity analysis was performed using TBtools software. The criteria for duplicate genes were: (1) the similarity between two aligned sequences was at least 70% with an e-value < 1e-10; (2) the length of the match covered at least 70% of the average length of two aligned sequences. Some of the genes identified by MCScanX as collinear were regarded as whole-genome duplication (WGD) genes, while other duplicates were classified based on their genomic proximity: those within the 100 kb region were defined as tandem duplication (TD), and those over 100 kb or located on different chromosomes were defined as dispersed duplication (DSD). Divergence time (T) was estimated using the formula T = Ks/(2r) Mya, where r = 7.44E−3 represents the synonymous substitution rate per site per million years (Chen et al., 2023b). The cis-regulatory elements (CREs) in the promoter sequences (upstream 2.0 kb) of the *ADH* genes were predicted using the PlantCARE software (Lescot et al., 2002) (http://bioinformatics.psb.ugent.be/webtools/plantcare/html/, accessed on 3 April 2024) and visualized by TBtools.

### 2.5 Phylogenetic and evolutionary analyses of *ADH* family

The multiple sequence alignment of ADH proteins was generated using MUSCLE 5.1 (Edgar, 2022), and the phylogenetic tree was constructed by the ML method using RAxML 8.2.12 under the PROTGAMMAJTTF model with 1000 bootstrap replicates (Edgar, 2022). Then the phylogenetic tree was visualized and modified using the online program ITOL (https://itol.embl.de/). Subsequently, the evolutionary history of *A. annua* and *A. argyi ADHs* were analyzed according to previous study (Kim et al., 2006). The nodes representing divergence points between the two species were identified based on two criteria: (1) the bootstrap value was higher than 50%; and (2) the relationship between the two species-specific clades was consistent with the species tree.

### 2.6 UV-B stress treatment and RNA-seq

Three genetically similar LQ-9 *A. annua* plants were exposed to UV-B irradiation (313 nm wavelength) using an illumination device operating at 40% of its maximum output power (80 W total capacity). Sampling was conducted at three time points following continuous UV-B irradiation: 0 h, 2 h, and 4 h. Fully expanded leaves were uniformly collected from three positions below the plant apex: 8 cm, 10 cm, and 15 cm. The experimental setup positioned the plant such that its apex was maintained 5 cm below the UV-B source. According to previous studies, samples under our post-treatment were immediately frozen in liquid nitrogen and stored at -80 °C in Eppendorf tubes (Eppendorf, Hamburg, Germany). Total RNA was extracted from *A. annua* samples with FastPure Plant Total RNA Isolation Kit (Vazyme). Qualified RNA was sequenced using 2 × 150 bp paired-end protocol on the Illumina NovaSeq 6000 platform.

### 2.7 *ADH* genes expression profile and WGCNA analysis

RNA-seq reads were mapped to LQ-9 haplotype 0 genome using HISAT2, and gene expression was calculated with StringTie (Pertea et al., 2016). Differentially expressed genes (DEGs) among different treatment were identified using DESeq2 (Wang et al., 2010), with |log_2_(fold change) ≥ 1 and p-value ≤ 1e-6. Publicly available RNA-seq datasets of *A. annua* under multiple treatments were obtained from NCBI (PRJNA435470 (SRP133983) and PRJNA601869) (Zhang et al., 2018) (Ma et al., 2020): Brassinosteroid (BR), Gibberellins (GA), light treatments, BR+UV-B, and GA+UV-B, and then these transcriptome data were mapped to the LQ-9 haplotype 0 genome. The co-expression network modules between various treatments and genes were constructed using the Weighted Gene Co-expression Network Analysis (WGCNA) package in R (Langfelder and Horvath, 2008).

### 2.8 Gene expression clustering and gene ontology enrichment analysis

The time trend analysis of DEGs was conducted using the Mfuzz 2.67.0 package in R, and the DEGs were classified into clusters (Kumar and Futschik, 2007). Gene ontology annotation was performed with eggNOG-mapper 2.1.5 (Cantalapiedra et al., 2021). GO enrichment analysis was carried out using clusterProfiler (Wu et al., 2021), and enrichment results with p-Value ≤ 1e-4 were retained.

### 2.9 PCR amplification of *AarADH* and genes expression validation of *AanADH*

Total RNA was extracted from fresh leaves with FastPure Plant Total RNA Isolation Kit (Vazyme). First-strand cDNA was synthesized with a HiScript III 1st Strand cDNA Synthesis Kit (+ gDNA wiper) (Vazyme, Nanjing, China) according to the manufacturer’s instructions. Primers for PCR and qRT-PCR listed in **Supplementary Table S1** were designed and synthesized by IGE Biotechnology Co., Ltd. (GuangZhou, China). In the *A. argyi ADH1-like* PCR amplification experiment, primers were designed on the second exon of the *Aar4B_1T00497.3* gene, and enzyme-free water was used in place of the positive control cDNA as a negative control. The PCR amplification conditions for the gene-specific primers were as follows: initial denaturation at 98 °C for 30 seconds, followed by 35 cycles of denaturation at 98 °C for 10 seconds, annealing at 55 °C for 15 seconds, extension at 72 °C for 10 seconds, and a final extension at 72 °C for 1 minute.

The expression of candidate *AanADH* genes was verified using qRT-PCR. The qRT-PCR reaction was performed using the Applied Biosystems ABI 7500 PCR System (ABI, United States). *β-actin* was used as a reference. The PCR amplification mixture contained 2 μl of cDNA, 10 μl of ChamQ Universal SYBR qPCR Master Mix (Vazyme Biotech Co., Ltd), 0.4 μl of 10 μM forward and reverse primers, and 7.2 μl ddH_2_O. The PCR reaction was performed with the initial denaturation step for 30 s at 95 °C; 40 cycles of 10 s at 95 °C and annealing at 60 °C for 30 s. The melting curve (60-95 °C) was used to check the specificity of each qRT-PCR reaction. The standard curves were generated using a two-fold dilution gradient of the cDNA. Amplification efficiency (E = 10-1/slope-1) and correlation coefficient (R2) values were calculated by standard curves. The relative gene expression was calculated using the 2–ΔΔCt method. Additionally, Tukey’s HSD test was employed to evaluate the differences of genes expression.

## 3 Results

### 3.1 Identification and physicochemical characteristics of *ADH* genes in *A. annua*

**Figure 1:**
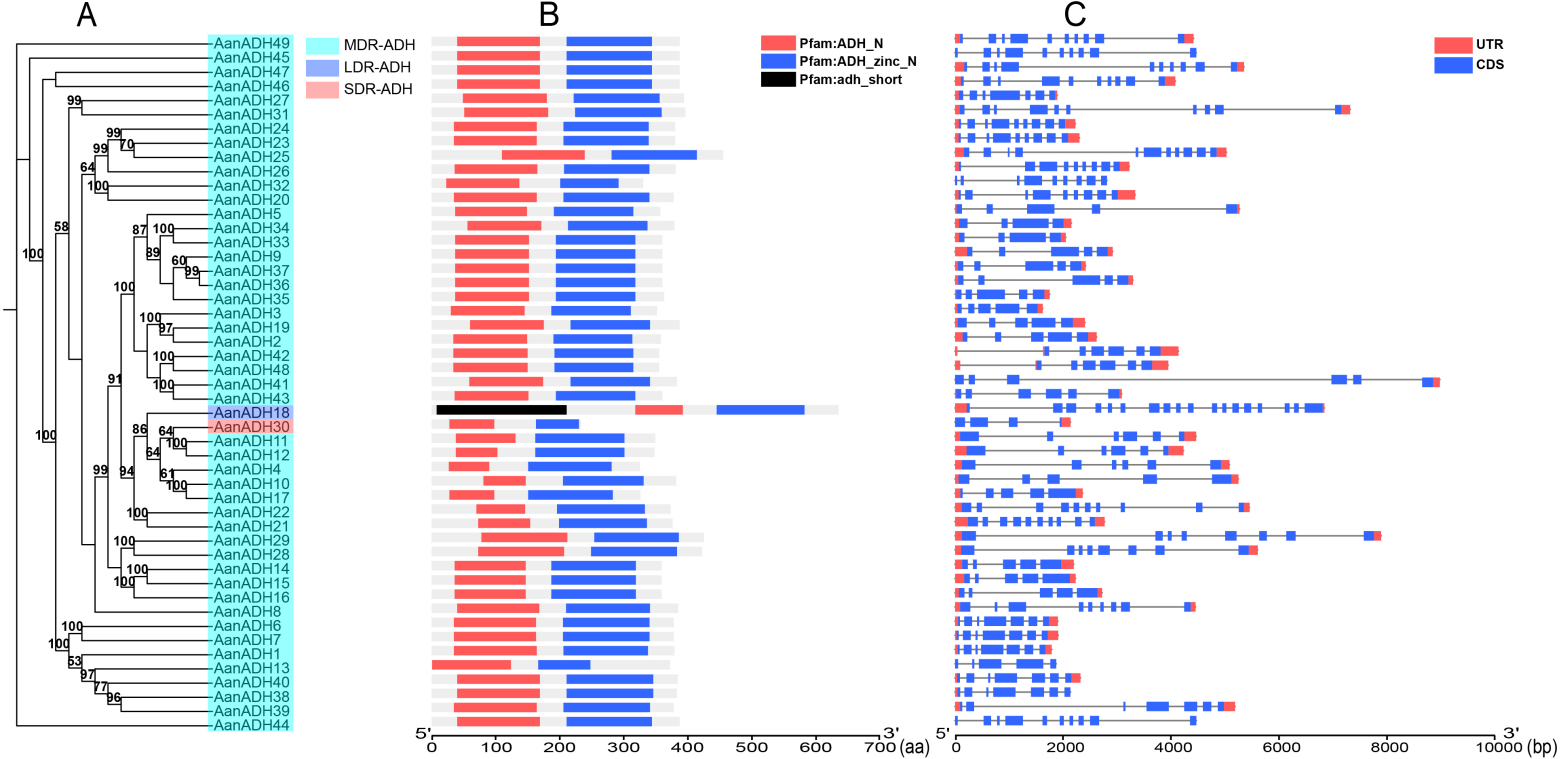
Information of *AanADH* genes. **(A)** Phylogenetic relationships (numbers on the nodes represent supporting values, with only values ≥ 50 displayed). **(B)** Domain information. **(C)** Gene structure.

A total of 49 *ADH* genes were identified in *A. annua* LQ-9 haplotype 0 genome, and were named as *AanADH1* to *AanADH49* (**Supplementary Table S2**). Analysis with HMMER (v3.1b2) revealed that all these genes possessed the structural domains ADH_N and ADH_zinc_N. Specifically, *AanADH18* contained an additional adh_short domain, resulting in a notably longer amino acid sequence compared to other members of the *AanADH* family (**Figures 1A, B**). The gene structure of *AanADH18* was verified using the genome browser IGV, and the results from IGV were consistent with the genomic prediction outcomes (**Figure 1C**; **Supplementary Figure S1**).

The number of exons varied from four (*AanADH23*, *AanADH33*, *AanADH34*) to 18 (*AanADH18*) (**Figure 1C**; **Supplementary Table S1**). The number of amino acids ranged from 233 (*AanADH30*) to 635 (*AanADH18*), representing all the subfamilies (SDR-ADH, MDR-ADH, and LDR-ADH) (**Supplementary Table S2**). AanADH proteins exhibited a theoretical pI that ranged from 4.66 (*AanADH30*) to 9.18 (*AanADH18*), and MW varied from 25.24 (*AanADH30*) to 68.39 kDa (*AanADH18*). Subcellular localization predictions showed that a majority AanADH proteins were located in cytoplasm, while minor located in mitochondria and *AanADH30* uniquely located in chloroplasts, and *AanADH20* was anticipated to be dual-localized, presenting in both cytoplasmic and mitochondrial compartments. Additionally, the majority of AanADH proteins were hydrophobic, with 35 exhibiting positive GRAVY values and 14 showing negative values, reflecting structural diversity (**Supplementary Table S2**).

### 3.2 Motifs analysis of 49 AanADH proteins

**Figure 2:**
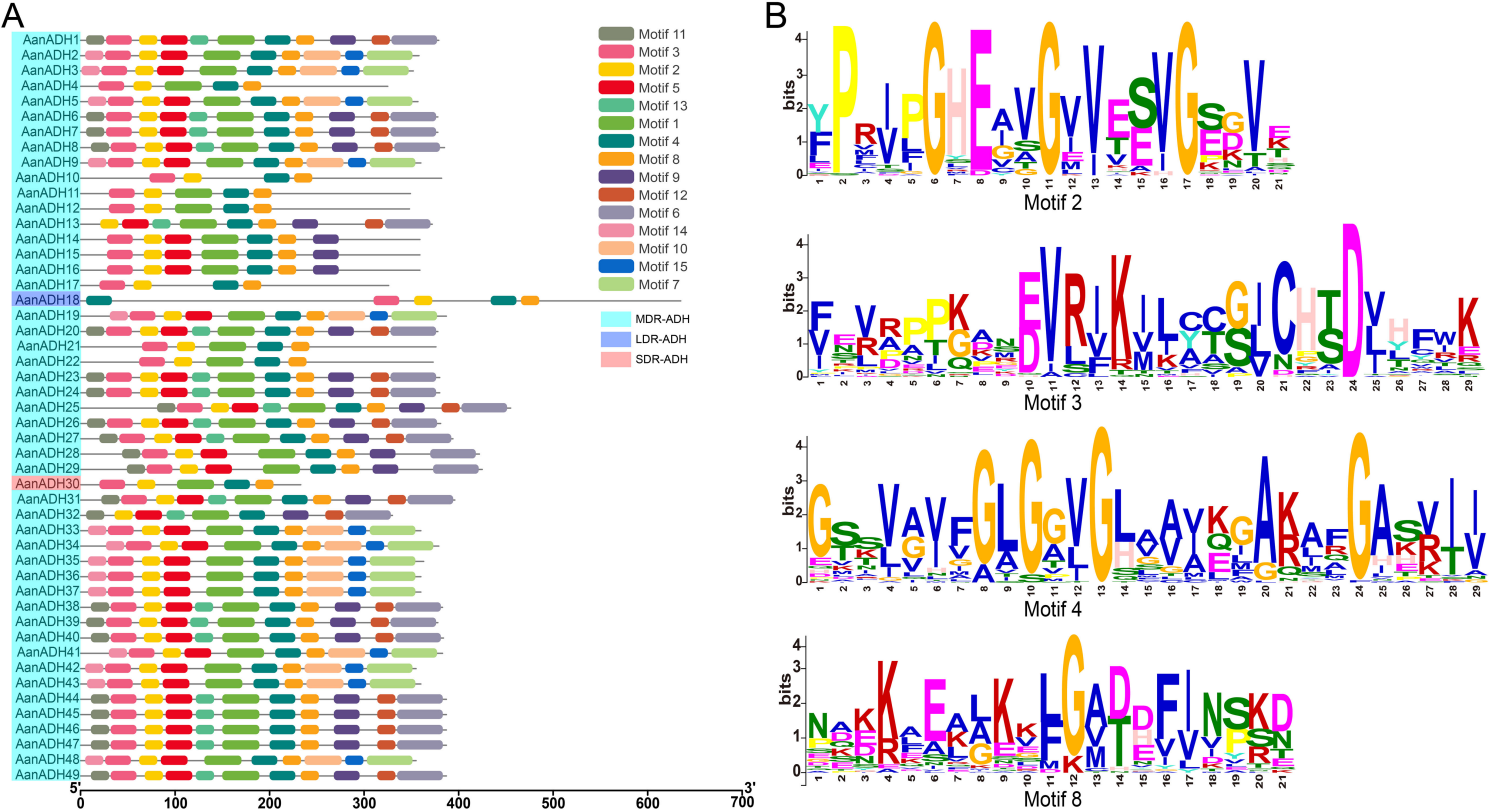
Motifs in AanADH proteins. **(A)** The motif composition of AanADH proteins. **(B)** Sequence analyses of conserved motifs.

To elucidate the conserved motifs within the AanADH protein family, the MEME online software was utilized to analyze 49 sequences of AanADH proteins. A total of 15 conserved motifs were identified (**Figure 2A**), in which motifs 2, 3, 4, and 8 were universally present (**Figure 2B**), and motif 5 occurred in 40 proteins, while motif 1 was absent only in AanADH18.

Furthermore, the ADH_N and ADH_zinc_N domains of 49 AanADH proteins were further compared. The ADH_N domain comprised 64 to 135 amino acids, while the ADH_zinc_N domain contained 68 to 140 amino acids. Numerous conserved sites were identified, consistent with AanADH motif conservation (**Supplementary Figure S2**), highlighting both diversity and uniformity in AanADH protein sequences.

### 3.3 Analyses of the chromosomal distribution, collinearity and gene duplication types of *AanADH* genes

**Figure 3:**
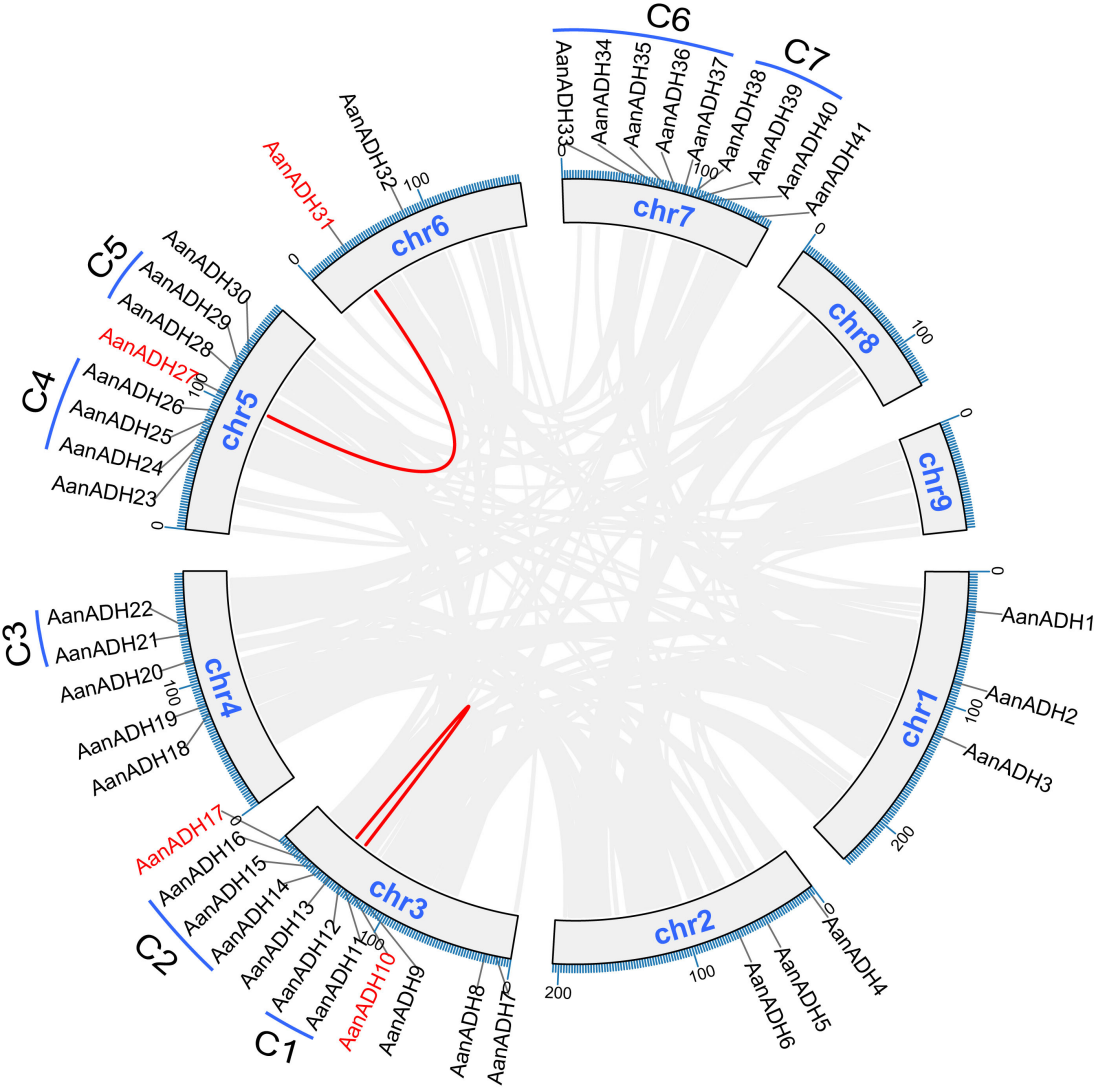
Collinearity analysis, chromosome distribution, and duplication types of the *AanADH* genes. Grey lines signify genes that have undergone WGD, while the red lines distinctly highlight the collinear *AanADH* genes. C1-C7 represent the seven tandem repeat gene clusters of the *AanADH* genes.

Genome annotation revealed 41 out of 49 *AanADH* genes unevenly across chromosomes one to seven in *A. annua*, absent on chromosomes eight and nine (**Figure 3**). Additionally, 60 duplicated gene pairs were identified within 49 *AanADH* genes (**Supplementary Table S2**), among which, two exhibited genomic collinearity (*AanADH10*-*AanADH17*, *AanADH27*-*AanADH31*), suggesting WGD (**Figure 3**). Notably, 26 TD and 32 DSD events were identified (**Supplementary Table S3**), and a distinct TD pattern was observed in 20 *AanADH* genes, forming eight gene clusters, with two to five genes each. Seven of these clusters were located on chromosome, while *AanADH47*-*AanADH48* clustered in a non-chromosomal region (**Figure 3**).

Ka/Ks analysis of 60 duplicated gene pairs revealed strong purifying selection (Ka/Ks < 1), with mean Ks > 0.5, indicating significant divergence. Duplication events occurred between 0.00 to 123.04 Mya (**Supplementary Table S3**). Thus, we hypothesize that the *AanADH* gene family has undergone significant purifying selection throughout its evolutionary.

### 3.4 Evolutionary differences of the *ADH* genes in *Artemisia* species

**Figure 4:**
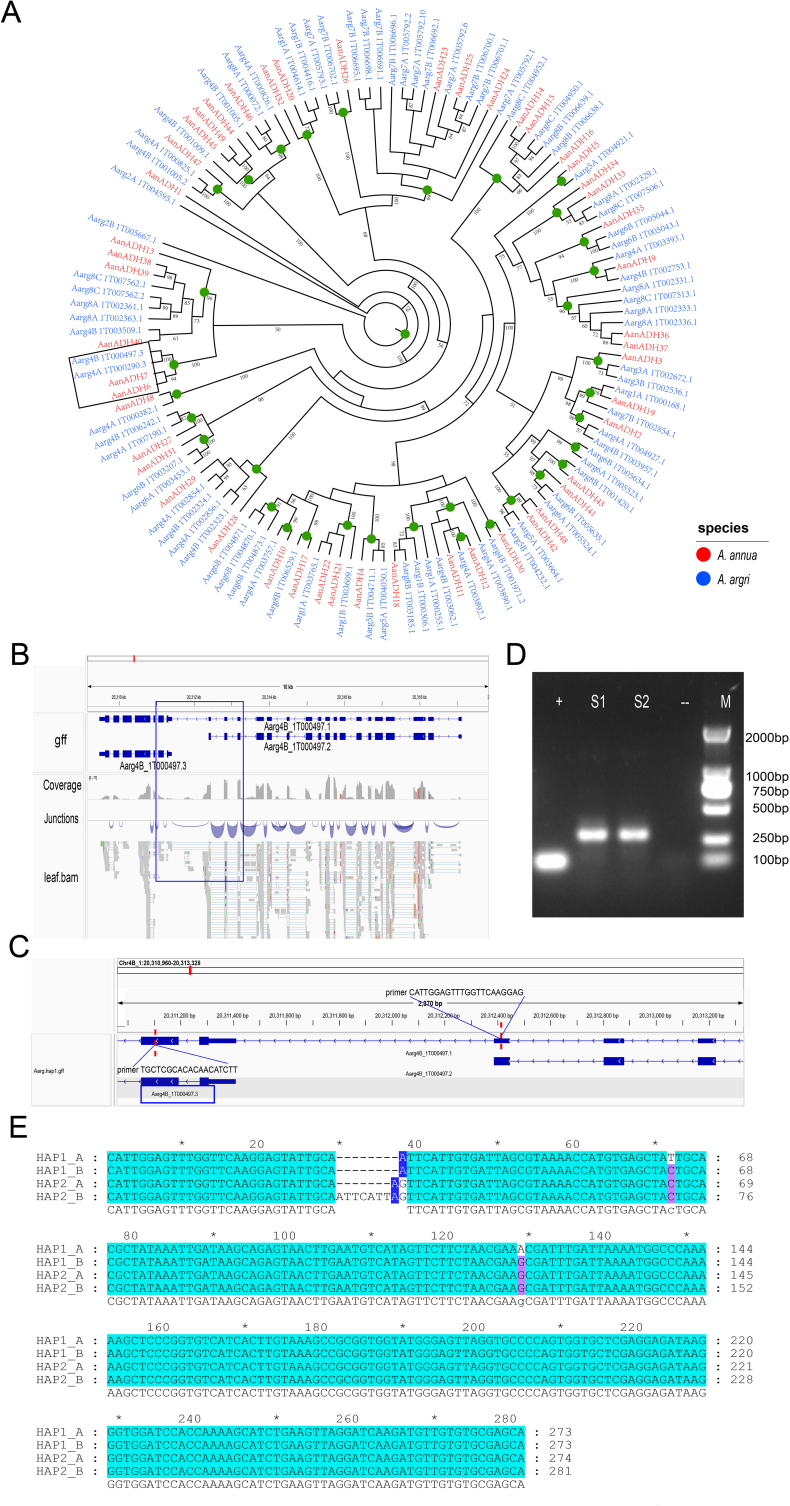
Evolutionary differences of the *ADH* genes in *Artemisia* species. **(A)** Phylogenetic tree of *Artemisia ADHs* from *A*. *annua* (Aan) and *A*. *argyi* (Aar). Branch numbers indicate bootstrap percentages ≥ 50%. *ADH* genes of different species are in different colors. Green circles denote the most recent common ancestor genes’ nodes. The branch outlined in black is the primary focus of our description. **(B)** The gene structure of *A*. *argyi ADH-like* and its gene fusion genes. The gene fusion event of *AarADH-like* is supported by transcriptome data. **(C)** Verification of the amplification fragment and primer sequences of the gene fusion event. **(D)** The agarose gel electrophoresis image of the *A*. *argyi ADH1-like* PCR Amplification Experiment. S1 and S2 represent the two replicate results of the amplified regions for gene fusion events, “+” indicates a positive control, and “-” indicates a negative control. **(E)** Multiple sequence alignment of the amplified region sequences of the four *ADH1-like* genes in *A*. *argyi.* HAP1 and HAP2 represent two haplotype genomes of *A*. *argyi*. “*” denotes odd multiples of 10 bases (e.g., for positions 10, 30, 50, ..., 270).

To explore the lineage-specific expansion of the *ADH* gene family in *Artemisia* species, 83 *ADH* genes in the *A. argyi* haplotype genome were identified, and a phylogenetic tree of *ADH* genes in *A. annua* and *A. argyi* was constructed. Nodes representing a divergence point and the most recent common ancestor (MRCA) of these two species were predicted (**Figure 4A**), and a total of 35 high-confidence nodes (≥ 50%) were identified. Comparative analysis revealed that the MRCA genes of *A. argyi* showed greater expansion than in *A. annua*, with no gene loss (**Supplementary Figure S4**). The expansion of *AanADH* genes was primarily driven by TD or DSD, resulting in the loss of two homologous genes, the gain of 16 homologous genes, and formation of 49 *AanADH* genes (**Figure 4A**; **Supplementary Figure S5**).

Syntenic analysis across *A. thaliana*, *A. annua*, and *A. argyi* revealed conserved synteny of *ADH* genes between *A. annua* and *A. thaliana*, showing evolutionary conservation of *ADH* gene family following the divergence of Asteraceae and Brassicaceae (**Supplementary Figures S6A, B**). Furthermore, *ADH* locus in *A. annua* frequently corresponded to two paralogous genes in *A. argyi*, further supporting that the expansion of *A. argyi ADH* gene family originated from a species-specific WGD event.

A unique evolutionary branch including *AanADH6*, *AanADH7*, *Aarg4A_1T00290.3* and *Aarg4B_1T000497.3* was identified (**Figure 4A**). Notably, *AanADH6* and *AanADH7* were involved in the biosynthetic pathway of artemisinin and named as *ADH1* gene. In *A. annua*, these genes expanded through dispersed duplications, while in *A. argyi*, they originated from a WGD event, which suggested that these genes may have distinct roles in *A. annua* and *A. argyi*, resulting in their distinct replication mechanisms. Furthermore, separate gene fusion events involving *Aarg4A_1T000290.3* and *Aarg4B_1T000497.3* with their respective neighboring genes were detected in the *A. argyi* genome. For instance, *Aarg4B_1T000497.3* merged with adjacent *Aarg4B_1T00497.2* to form the novel *Aarg4B_1T000497.1* (**Figure 4B**), which was validated by PCR (**Figures 4C, D**). Genomic analysis further revealed four highly similar *Aarg4B_1T00497.1* fusion copies across *A. argyi* HAP1 and HAP2 genomes. Multiple sequence alignment of the four *Aarg4B_1T00497.1* copies revealed significant insertions and deletions (indels). In the Sanger sequencing results, multiple nested-peak was observed (**Supplementary Figures S7A, B**), consistent with coexisting gene variants harboring indels and nucleotide polymorphisms. To clarify the specific variation patterns among these copies, TA cloning-based single-clone sequencing was performed, which confirmed *ADH1-like* gene sequence polymorphism in *A. argyi* and validated the occurrence of gene fusion events (**Supplementary Figure S7C**).

### 3.5 *AanADH* genes promoter regions cis-acting elements and WGCNA analysis

**Figure 5:**
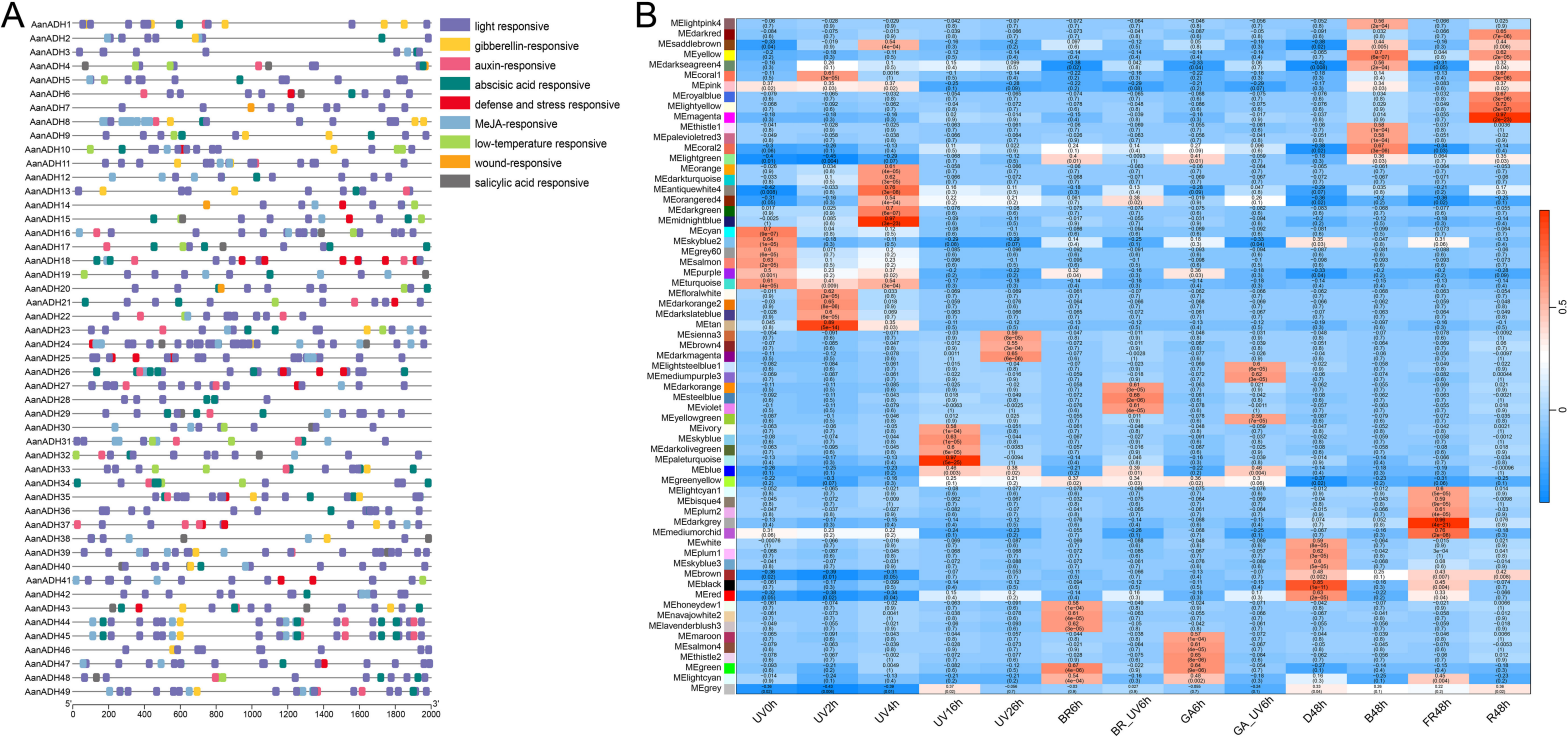
*AanADH* promoter regions CREs and WGCNA analysis. **(A)** CREs in *AanADH* promoter regions. **(B)** Module-trait associations. UV, ultraviolet irradiation b; BR, Brassinolide; GA, gibberellin; BR_UV, a combination of BR and UV-B; GA_UV, a combination of GA and UV-B; D, darkness; B, blue light; FR, far red light; R, red light.

To elucidate the potential biological functions of the *AanADH* genes, CREs within 2000 bp upstream regions of these genes was analyzed using plantCARE. The CREs related to stress, phytohormone, growth and development were identified, with significant enrichment of light-responsive elements (**Figure 5A**), implicating *AanADH* genes may involve in *A. annua* photoresponse regulation.

To assess the role of *AanADH* genes in light response, short-term UV-B treatment (0 h, 2 h, 4 h) was conducted on *A. annua* and transcriptome sequencing was performed. A total of 4.18 to 6.70 GB raw data was obtained per sample (**Supplementary Table S4**), with high correlation among replicate samples (**Supplementary Figure S8**). Besides, published transcriptomic data of long-term light exposure and hormone treatment of *A. annua* were downloaded and integrated with our data for genome-wide WGCNA analysis. The study classified 54,988 genes into 65 distinct co-expression modules, with *AanADH* genes predominantly enriched in the ‘turquoise’, ‘blue’, ‘brown’, ‘yellow’ and ‘greenyellow’ modules (**Supplementary Table S5**). Strikingly, *AanADH* expression strongly correlated with the following light treatments: UV0h, UV2h, UV4h, UV16h, UV26h, B48h, R48h, and D48h (**Figure 5B**). The ‘turquoise’ module, most enriched for *AanADH* genes, showed highly correlated with early UV-B treatments (UV0h, UV2h, UV4h) and harbored light-responsive transcription factors (WRKY, bHLH, MYB, bZIP, and BBX), which suggested *AanADH* expression was dynamically regulated by light, particularly UV-B, and had a complex response regulatory mechanism.

### 3.6 The expression pattern of *AanADH* genes under varying durations of UV-B stress

**Figure 6:**
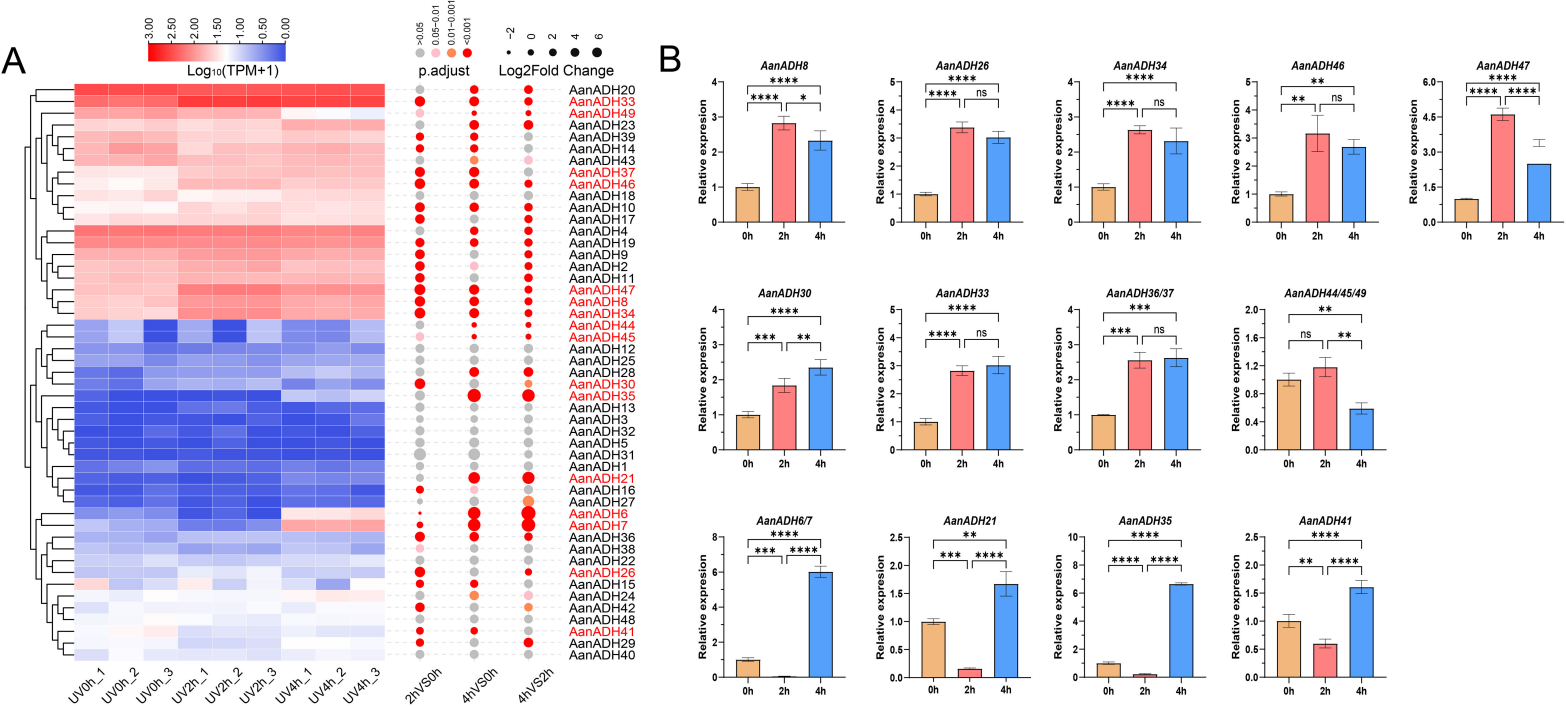
The expression of *ADH* genes in *A*. *annua*. **(A)** The expression heatmap of 49 *AanADH* genes after ultraviolet treatment at 0 h, 2 h and 4 h, the color scale represented the range of Log_10_ (TPM+1). 16 differentially expressed *ADH* genes are marked in red. **(B)** Relative expression levels of 16 DEGs for *ADH* in *A. annua* under different durations of UV-B treatment. The error bars represented the SDs. NS' indicates no significance;'*' indicates P <0.05; '**' indicates P < 0.01; '***' indicates P < 0.001; '****' indicates P < 0.0001. 0 h, 2 h and 4 h represent the three time points of UV-B treatment, respectively.

After brief UV-B irradiation treatments at 0, 2 and 4 h, respectively, the expression levels of genes showed noticeable changes at various time points. Differential expression analysis revealed that compared to UV0h, 1940 genes were significantly upregulated and 1797 genes significantly downregulated at UV2h, and 2253 genes significantly upregulated and 1602 genes significantly downregulated at UV4h, and compared to UV2h, 1636 genes significantly upregulated and 1136 genes significantly downregulated at UV4h. A total of 5961 DEGs were identified. DESeq2 analysis results revealed that 16 *AanADH* genes exhibited three distinct expression patterns under UV-B treatment: *AanADH8*, *AanADH26*, *AanADH34*, *AanADH46*, *AanADH47*, and *AanADH44*, *AanADH45*, and *AanADH49* showed an increase followed by a decrease; *AanADH30*, *AanADH33*, *AanADH37* displayed continuous upregulation; and *AanADH6*, *AanADH7*, *AanADH21*, *AanADH35* exhibited a decrease followed by an increase, which were consistent with the trends shown in the gene expression heatmap (**Figure 6A**). 5961 DEGs were grouped into eight clusters by clustering analysis, with differentially expressed *AanADH* genes mainly enriched in Cluster 1, Cluster 4, and Cluster 8 (**Supplementary Figure S9**). GO enrichment analysis revealed that genes in Cluster 1 were associated with cell cycle regulation and chloroplast function, genes in Cluster 4 were linked to light signaling and plant defense responses, and genes in Cluster 8 were involved in cell membrane structure, substance transport, cell growth regulation, and environmental stress responses (**Supplementary Figure S10**).

qRT-PCR experiment was conducted to verify the expression levels of the differentially expressed *AanADH* genes (**Figure 6B**). Due to high coding sequence similarity among the following gene pairs: *AanADH6*/*AanADH7*, *AanADH44*/*AanADH45*/*AanADH49*, and *AanADH36*/*AanADH37* (though *AanADH36* was not among the DEG identified previously), primers targeted conserved regions shared across these genes were designed. Overall, *AanADH* genes exhibited diverse expression changes under UV-B stress. Specifically, *AanADH6/7*, *AanADH21*, and *AanADH35* showed significantly reduced expression levels at UV2h but increased at UV4h, while *AanADH8*, *AanADH26*, *AanADH30*, *AanADH33*, *AanADH44*, *AanADH36/37*, *AanADH46*, and *AanADH47* were significantly upregulated. These results suggested that these genes may enhance *A. annua*’s adaptability to UV-B irradiation stress.

## 4 Discussion

*ADH* gene family members are widely distributed in eukaryotes and prokaryotes (Strommer, 2011). With the development of genome sequencing technology, a series of *ADH* genes or *ADH-like* genes have been identified in *Poaceae*, *Rosaceae*, *Brassicaceae*, *Fabaceae*, and *Pinaceae* plants (Thompson et al., 2010). A large number of studies on the characteristics of *ADH* genes have linked it to both biotic and abiotic stresses (Su et al., 2020; Shen et al., 2021; Wang et al., 2024), greatly enhancing our understanding of various plant responses (Zeng et al., 2020; Shen et al., 2021). However, the *ADH* gene family has not been reported in *A. annua*. In this study, a total of 49 *AanADH* genes were identified, including one long-chain genes, one short gene, and the remaining 47 medium-chain genes. The number of *AanADH* genes is more than maize (seven members), wheat (22 members), tomato (36 members), and *A. thaliana* (44 members), but less than in tobacco (53 members), Chinese white pear (68 members), apple (82 members) (Wang et al., 2024; Zeng et al., 2020), and *A. ariyi* (83 members).

Generally, the evolution of gene families largely depends on the organization of gene structure. The *AanADH* genes were characterized by long genomic sequences but few amino acids, and the amino acid sequence length of most members was concentrated between and 400 amino acids. Additionally, significant variations were observed in the exon/intron structure and protein motif composition among the 49 *AanADHs.* The coexistence of universally conserved and uniquely variable motifs highlighted the intricate balance between conservation and diversity among AanADH proteins.

The diversity of plant genes relies on events of gene duplication, a process that is a key evolutionary mechanism for the expansion of gene families and provides the possibility for the divergence of gene functions (Das et al., 2016; Das Laha et al., 2020). Duplicated gene pairs were identified in *AanADHs* family, 58 out of 60 duplicated gene pairs were driven by TD and DSD. The prevalence of TD in the *AanADH* genes further substantiates the established pattern of high tandem repeatability characteristic of *ADH* gene family. Additionally, all duplicated gene pairs exhibited Ka/Ks values less than 1, suggesting that these genes have been subjected to purifying selection, which may be related to the relatively stable function of this gene family in responding to abiotic stress.

*A. annua* and *A. argyi*, as representative species of the genus *Artemisia*, have exhibited significant genomic differences, particularly with the unique WGD event that occurred in the *A. argyi* genome. The *ADH1* gene, playing a crucial role in the biosynthesis of artemisinin, exists in two copies in both *A. annua* and *A. argyi*, showed different duplication mechanisms between the two species. In *A. argyi*, the whole-genome duplication has led to a reduction in intergenic distances, with *ADH1-like* genes being less than 1 kb away from neighboring genes, thereby promoting gene fusion events between *ADH1-like* genes and neighboring genes. In contrast, in *A. annua*, the distance between *ADH1* genes and the aforementioned neighboring genes exceeded 200 kb, and there are more than 20 other genes present between them that no gene fusion events were observed. This discovery not only confirmed the dynamism of the *Artemisia* genus’ genomes but also suggested their potential to continuously generate new genes, which was important for understanding the adaptive evolution and genomic diversity of these species.

Plants, when exposed to stressors such as intense light, UV radiation, and changes in the light cycle, may produce excessive reactive ROS (Chen et al., 2022). This overproduction can adversely affect photosynthesis, metabolism, and overall growth and development (Muhammad et al., 2020). *ADH* genes play a crucial role in the plants’ response to various biotic stresses, particularly oxidative stress (Muhammad et al., 2020; Strommer, 2011). The activation of *ADH* genes may enhance the plants’ antioxidant capacity, thereby protecting plant cells from ROS-induced damage (Strommer, 2011; Jin et al., 2016; Su et al., 2020). Previous studies have primarily focused on the role of *ADH* genes in response to abiotic stresses such as flooding, low temperature, drought, and salinity, as well as their key roles in the synthesis of volatile esters. For instance, Shen et al. speculated that *TaADH1/2*, *TaADH3* and *TaADH9* played an important role in waterlogging stress, which was an important basis for screening waterlogging tolerant wheat varieties (Shen et al., 2021). Noguchi et al. found that low temperature stress (5 °C, 7.5 °C, 10 °C) can significantly improve the activities of *ADH* and *PDC* in rice seedlings, and enhanced the ethanol fermentation pathway (Kato-Noguchi and Yasuda, 2007). *ADH* genes play role in promoting alcohol production at later stages in pear fruit development (Zeng et al., 2020). However, there are relatively few reports on how *ADH* genes respond to light stress, especially UV-B stress.

In this study, we have conducted an in-depth analysis of gene expression patterns of *A. annua* at different time points of UV-B irradiation (0 h, 2 h and 4 h). A total of 5961 DEGs were identified and clustered into eight clusters, with *AanADH* genes predominantly found in Cluster 1, Cluster 4, and Cluster 8. According to previous studies, genes that showed a continuous expression increase under UV-B stimulation were primarily responsive to the cumulative damage of UV-B irradiation or the direct response to UV-B signals. Genes that exhibited an initial increase followed by a decrease in expression may be activated by UV-B radiation at the initial stage and then inactivated due to some mechanism. As for genes that first decreased and then increased in expression, it was possible that the strong UV-B stimulus initially suppressed their expression, but over time, certain activation mechanisms were triggered, leading to a gradual increase in their expression levels. GO enrichment analysis revealed a high degree of consistency between gene expression trends and enrichment outcomes, indicating that *AanADH* genes were likely regulated by UV-B irradiation, and qRT-PCR confirmed their dynamic expression patterns. These findings reinforced our understanding of gene expression dynamics and laid the groundwork for further research into *A. annua*’s UV-B stress response. Additionally, previous studies have indicated that the *ADH1* gene, which plays a role in the biosynthesis of artemisinin, experiences a significant upregulation in expression after extended periods of UV-B radiation (Ma et al., 2020). However, our research has uncovered a distinct expression pattern for the *ADH1* gene under shorter UV-B treatment durations. Specifically, following UV2h treatment, the gene’s expression level was approximately 15 times lower compared to the untreated control. In contrast, after UV4h treatment, there was a marked increase in expression level, which was about five times higher than that of the untreated control and around 105 times higher than the level observed after UV2h treatment. These results suggested that the expression pattern of the *ADH1* gene in *A. annua* under UV-B stress was likely governed by intricate molecular regulation, necessitating further in-depth research to elucidate the specific mechanisms involved.

## 5 Conclusions

In this study, we conducted the first comprehensive and systematic analysis of the *ADH* gene family in *A. annua*. A total of 49 and 73 *ADH* genes were identified in the *A. annua* genome and *A. argyi* genome, respectively. The phylogenetic relationships, gene structures, conserved motifs, and cis-elements of the *AanADH* genes were analyzed by bioinformatics analyze. The phylogenetic relationship between *A. argyi* and *A. annua ADH* genes indicated that these genes have undergone substantial gene duplication events within each species after their most recent common ancestor, leading to a significant difference in the number of *ADH* genes between the two species. Specifically, *ADH1*, crucial for artemisinin production, had two copies in both species, expanding through via DSD in *A. annua* but WGD in *A. argyi*. Meanwhile, we identified and validated gene fusion events of *ADH1-like* genes in *A. argyi*, indicating that the *Artemisia* genus genome remains dynamic and capable of generating new genes. The CREs and WGCNA results showed that the promoters of *AanADH* genes contained a large number of light-responsive cis-elements, and the expression of these gene family members was regulated by various types of light treatments. The expression patterns of the *AanADH* genes under UV-B light exposure at 0 h, 2 h, and 4 h indicated that these genes were regulated in response to UV-B stress and participated in the adaptive response of *A. annua* to UV-induced stress through multiple pathways. Our research provided valuable references for the study of *AnaADH* genes responds to abiotic stress and development responses.

## Data Availability

The raw RNA-seq data information can be accessed from Global Pharmacopoeia Genome Database at http://www.gpgenome.com/species/92 (accession numbers 55-64). Data supporting the findings of this work are available within the paper and its supplemental information files. The datasets generated and analyzed during the study are available from the corresponding author upon reasonable request.
