# Supplementary material for: Genome-wide identification and expression analysis of the ADH gene family in Artemisia annua L. under UV-B stress
Source: Front Plant Sci. 2025 Mar 19;16:1533225. doi: 10.3389/fpls.2025.1533225 (PMC11961895; doi:10.3389/fpls.2025.1533225)
Supplement: Supplementary file 2 [file DataSheet1.docx]

**
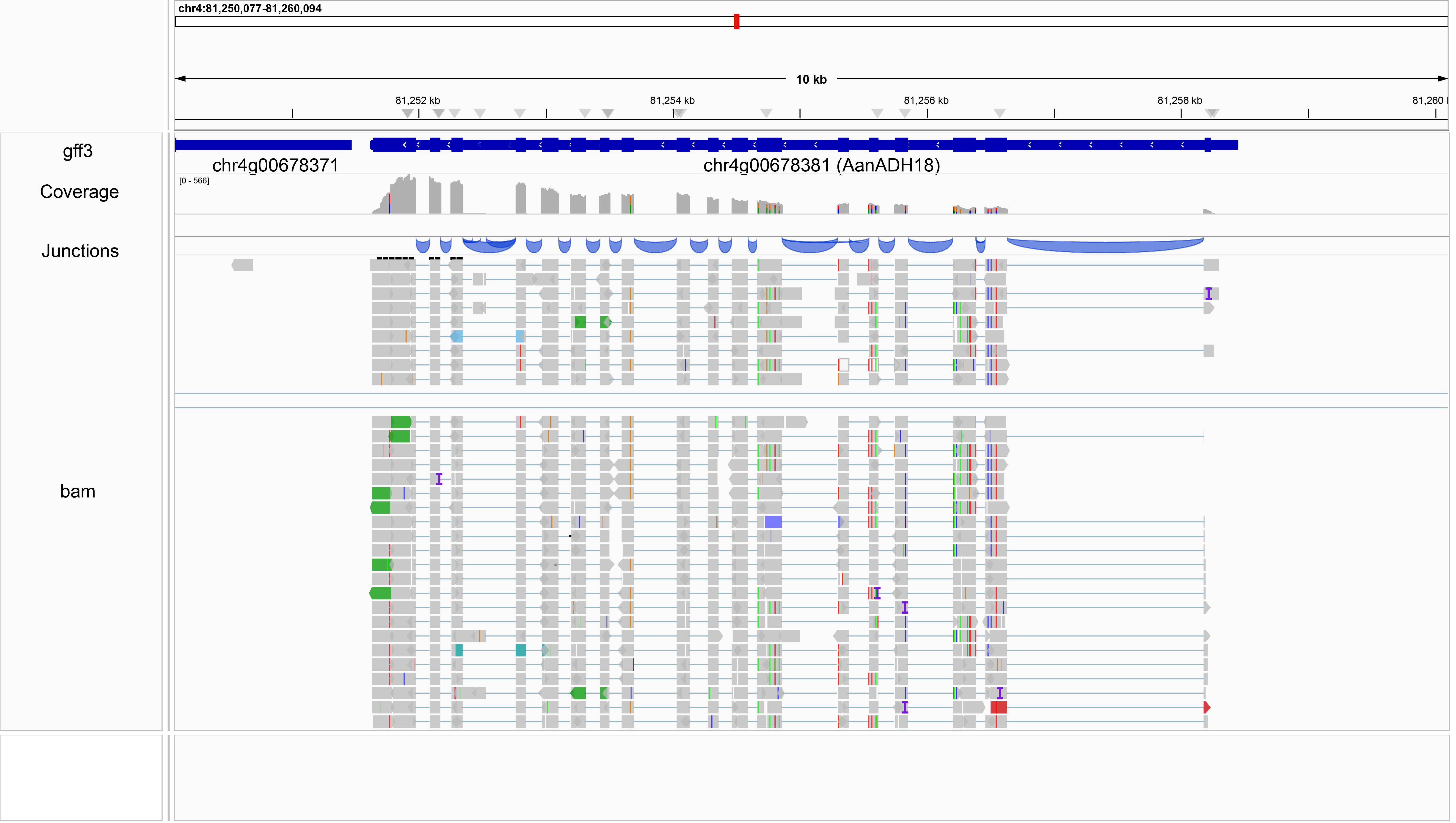
**

**Supplementary Figure 1** Alignment of *AanADH18* Gene Structure with Transcriptome Data.


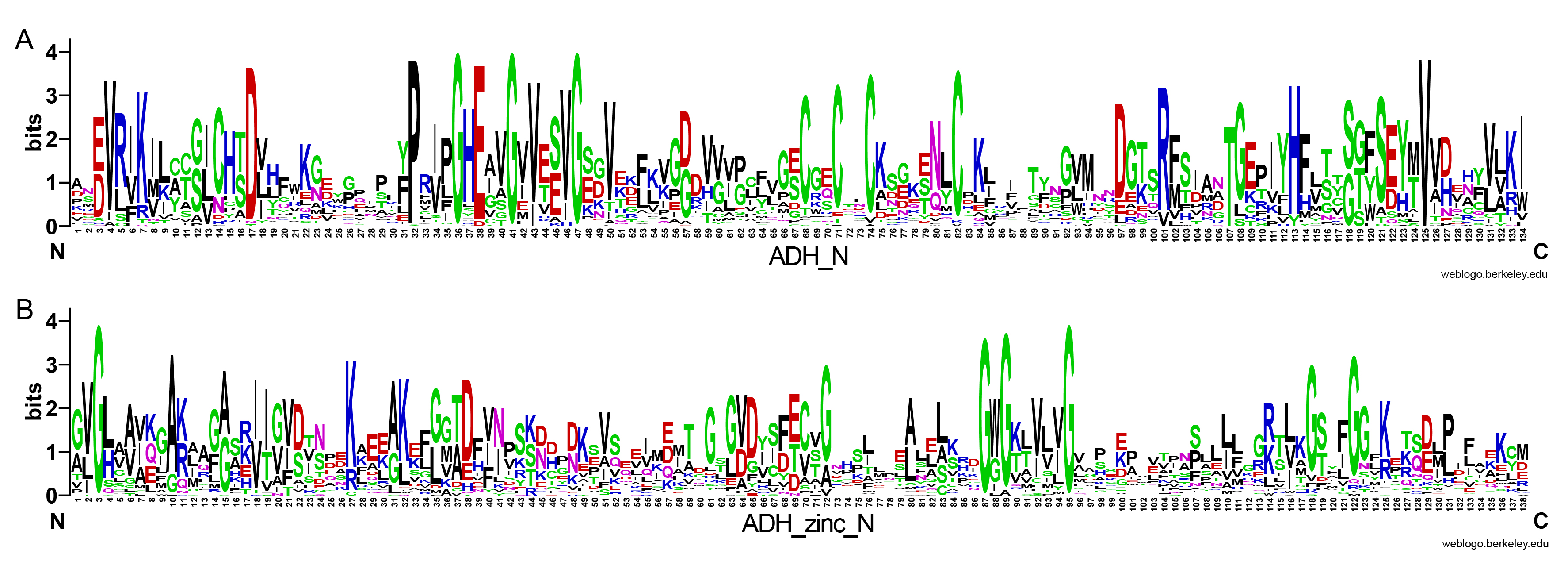


**Supplementary Figure 2** WebLogos of the conserved structural domains of the *ADH* family members in *A. annua*. **(A)** ADH_N domain. **(B)** ADH_zinc_N domain


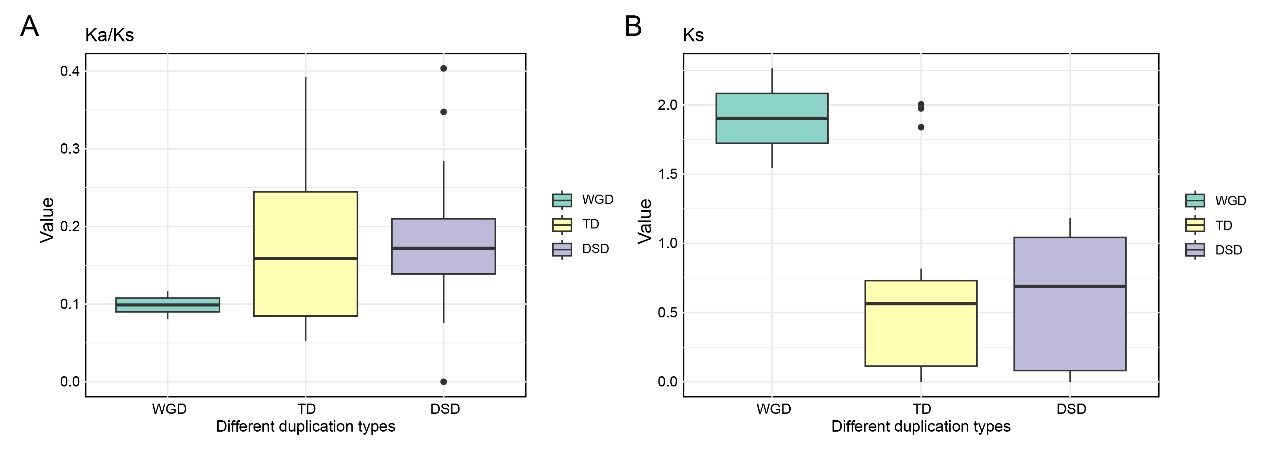
: **Supplementary Figure 3** Evolutionary selection pressures on *AanADH* gene pairs. **(A)** The Ka/Ks ratios of 60 duplicated gene pairs. **(B)** The Ks values of 60 duplicated gene pairs.

*
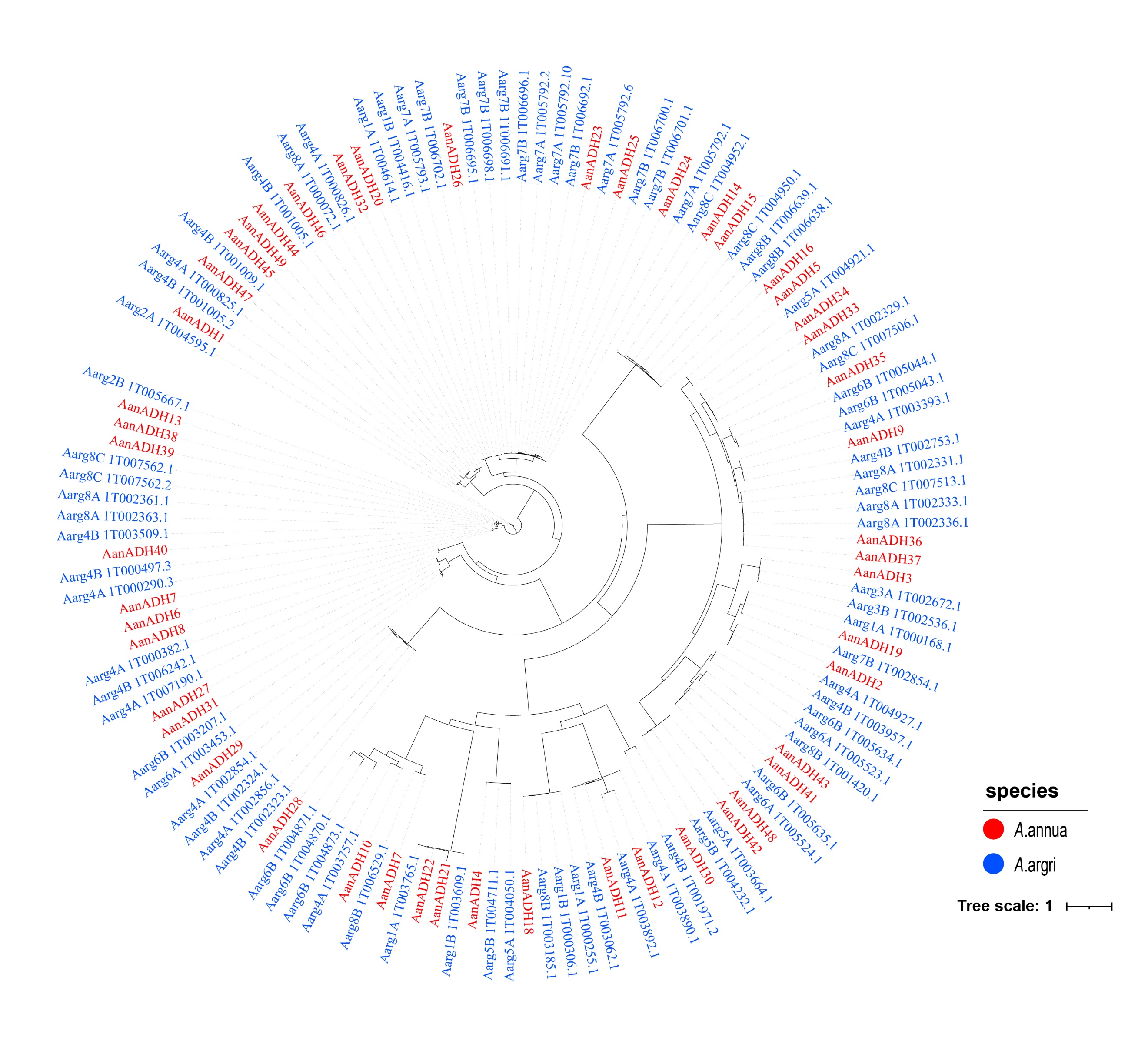
*

**Supplementary Figure 4** Conserved branch length phylogenetic tree of *ADH* Genes from *A. annua* and *A. argyi.*

*
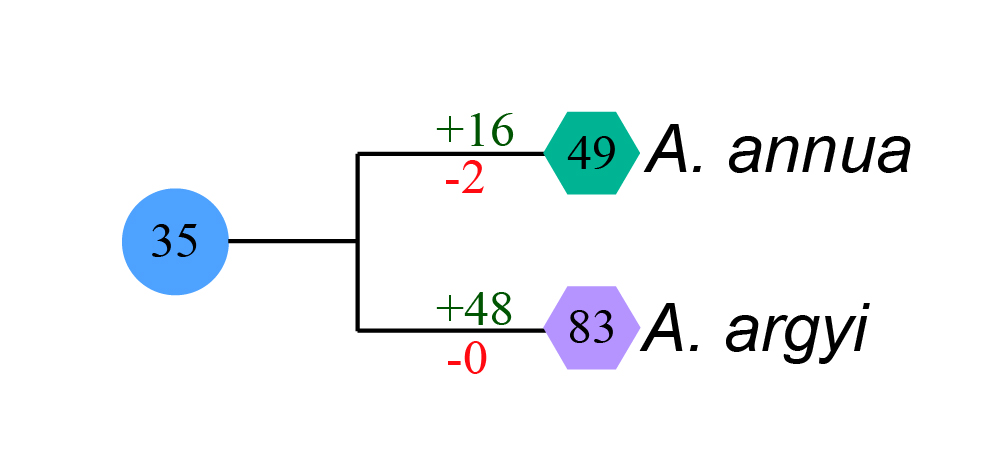
*

**Supplementary Figure 5** Copy number variation of *ADHs* in *A. annua* and *A. argyi*

*
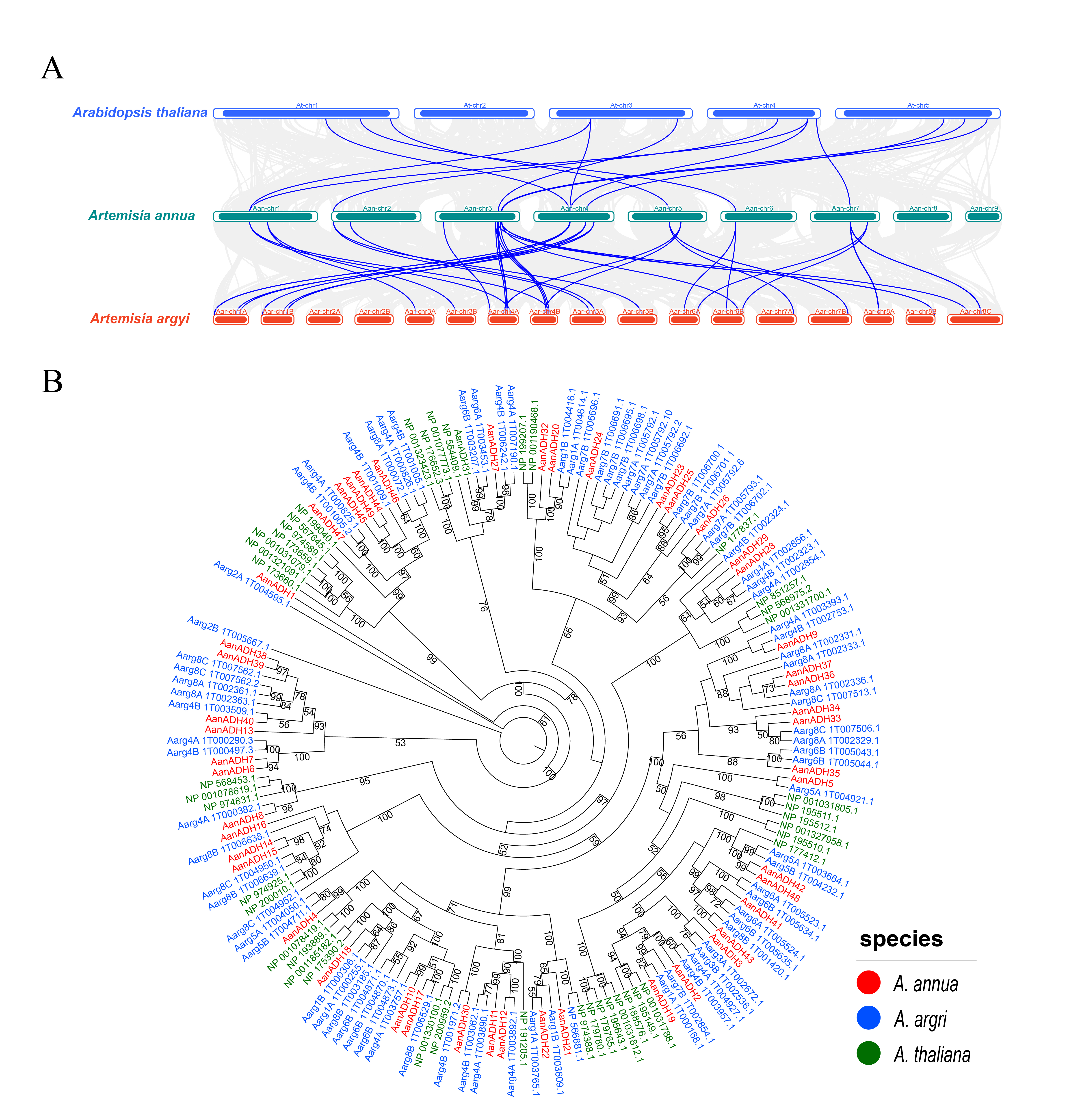
*

**Supplementary Figure 6** Evolutionary relationships of *ADH* genes among *A. thaliana, A. annua, and A. argyi*. **(A)**. Synteny analysis. **(B)**. Phylogenetic tree.

*
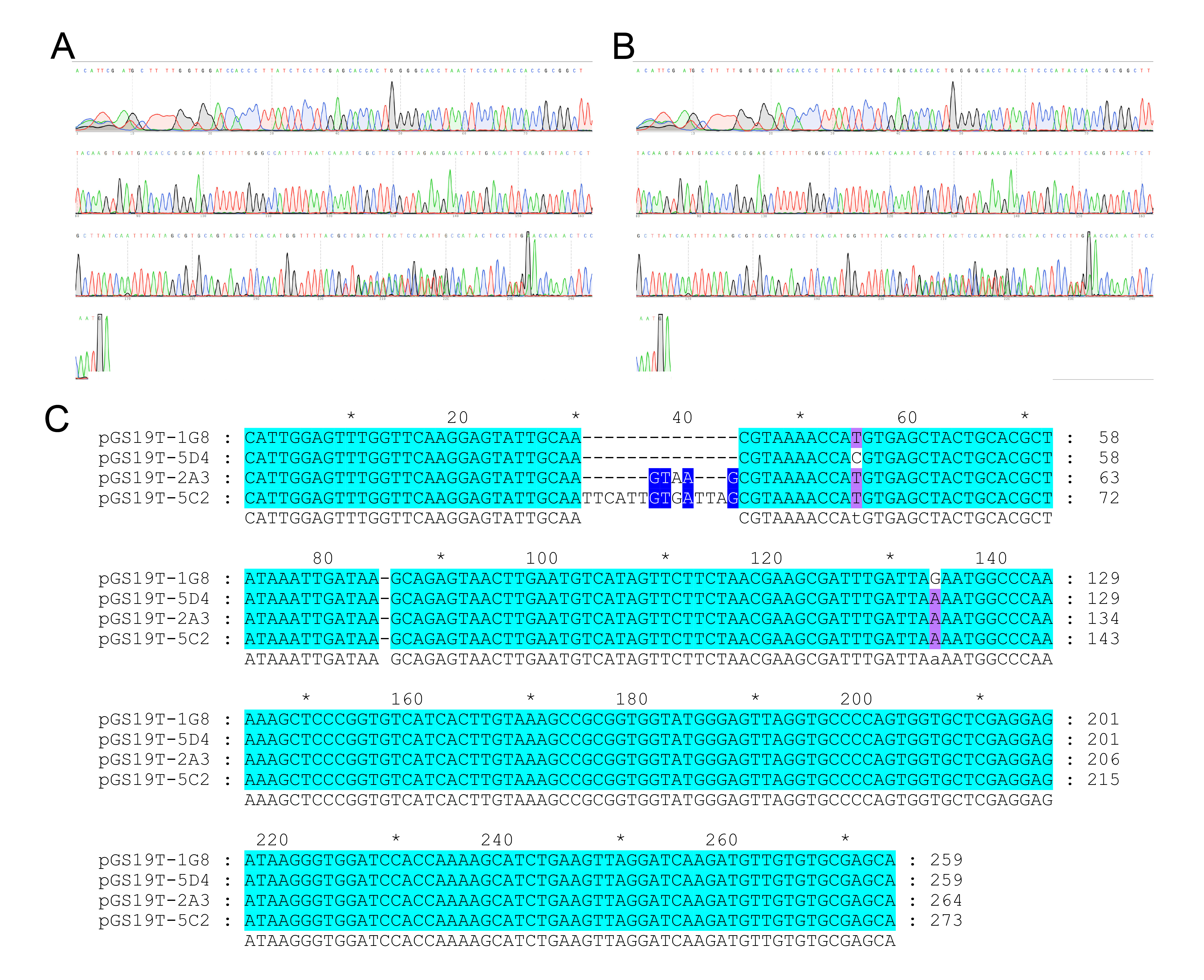
*

**Supplementary Figure 7** Sanger sequencing electropherogram of the *ADH1-like* gene fusion amplification product in *A. argyi*. **(A)** Forward sequencing. **(B)** Reverse sequencing. **(C)** Multiple sequence alignment of TA cloning sequencing

**
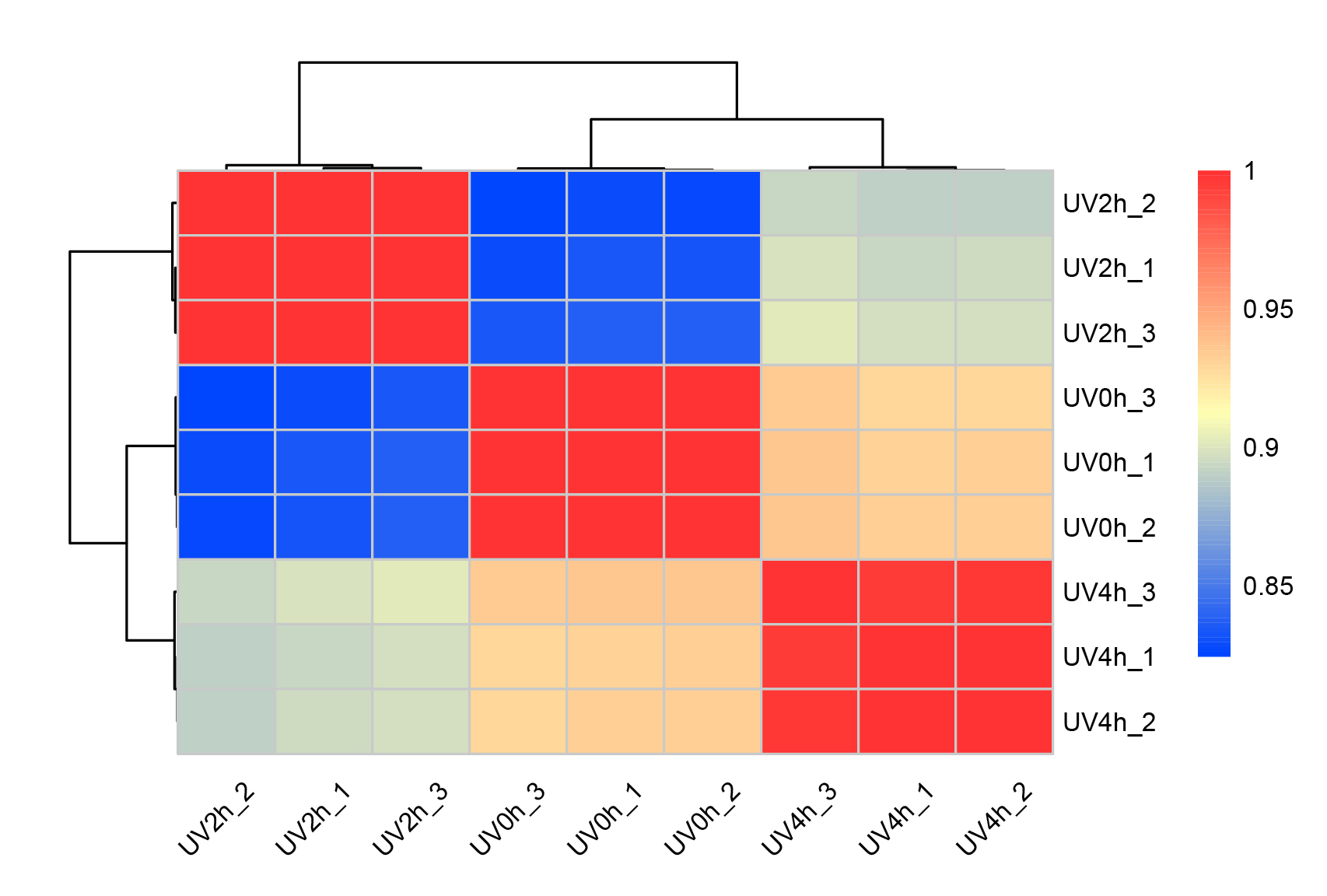
**

**Supplementary Figure 8** Correlation of transcriptome data under ultraviolet light treatment of varying durations.


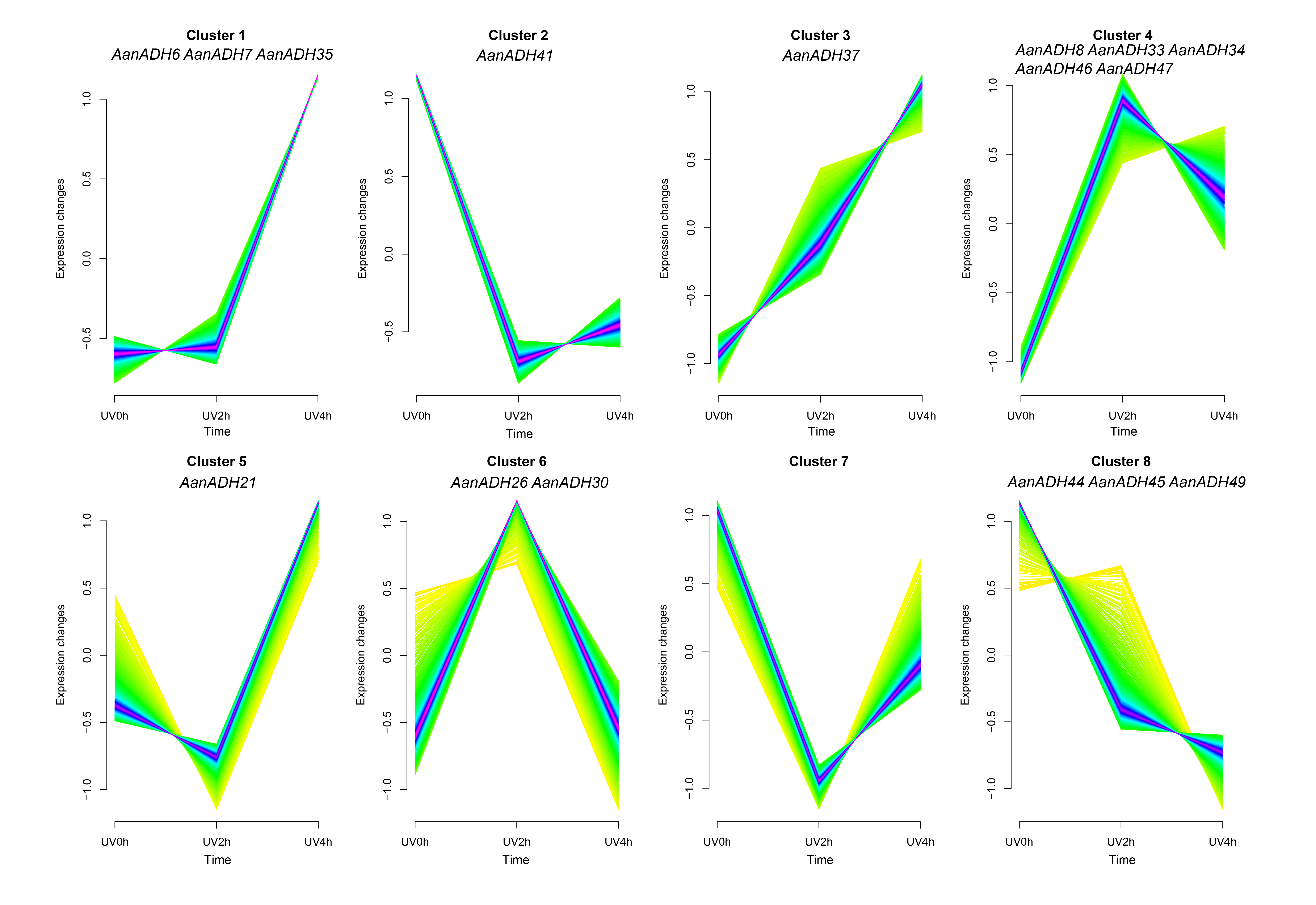


**Supplementary Figure 9** Expression cluster of differentially expressed genes across various ultraviolet treatment times.

**
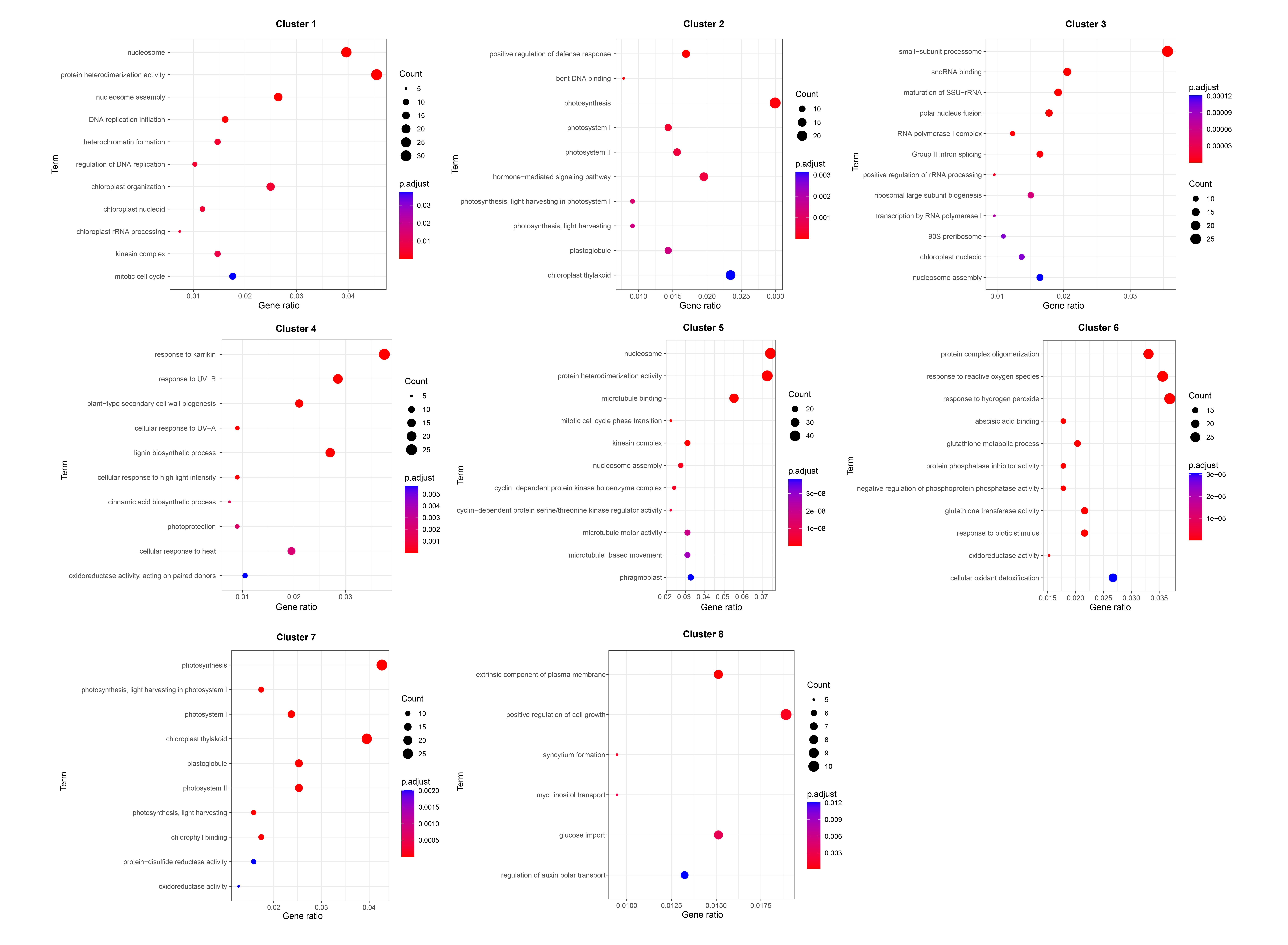
Supplementary Figure 10** GO enrichment analysis of clustered genes.
